# Low serum apelin levels are associated with mild cognitive impairment in Type 2 diabetic patients

**DOI:** 10.1186/s12902-022-01051-1

**Published:** 2022-05-24

**Authors:** Yongli Jiang, Shidi Wang, Xinghui Liu

**Affiliations:** 1 Department of Clinical Laboratory, Shanghai Pudong New Area Geriatric Hospital, Pudong New Area, Shanghai, 200135 China; 2 Department of Clinical Laboratory, Shanghai Gongli Hospital, Second Military Medical University, Pudong New Area, Shanghai, 200135 China

**Keywords:** Type 2 diabetes mellitus, Mild cognitive impairment (MCI), Apelin

## Abstract

**Background:**

Apelin is a new adipokine that is secreted by adipocytes, and is associated with insulin resistance (IR), inflammation, and obesity. This study was designed to investigate the role of apelin in type 2 diabetes mellitus (T2DM) patients with mild cognitive impairment (MCI).

**Methods:**

A total of 235 patients with T2DM were included. The cognitive function of patients was evaluated using Montreal Cognitive Assessment (MoCA) tool, then patients were divided into MCI group and non-MCI group according to the MoCA score. Blood sample was analyzed for the level of apelin by enzyme-linked immunosorbent assay (ELISA).

**Results:**

The MCI group (*n* = 73) presented lower serum apelin levels compared with the patients with normal cognitive function (*P* < 0.001). Apelin levels showed significantly negative correlation with diabetes duration, triglyceride (TG), total cholesterol (TC), low-density lipoprotein cholesterol (LDL-C, creatinine and high sensitivity C-reactive protein (hs-CRP), and positive correlation with high-density lipoprotein cholesterol (HDL-C) and brain-derived neurotrophic factor (BDNF). Multivariable logistic regression analysis indicated that serum apelin (OR = 0.304, 95%CI: 0.104–0.886, *P* = 0.029), as well as education levels, diabetes duration, cardiovascular disease, serum HbA1c, HDL-C, creatinine, and BDNF, were independent risk factors of MCI in patients with T2DM.

**Conclusions:**

Serum apelin level is reduced in T2DM patients with MCI. Apelin might has protective effect against cognitive impairment and serve as a serum biomarker of T2DM.

## Introduction

Type 2 diabetes mellitus (T2DM) is a metabolic disorder and associated with insulin resistance (IR), inflammation, and obesity. It can cause severe multi-systemic dysfunction, such as kidney, eye, peripheral, central nervous system, etc. A significant number of epidemiological studies establish that T2DM is a risk factor for cognitive impairment, dementia, and Alzheimer's disease [1–3]. Mild cognitive impairment (MCI) is a minor but obvious shift in memory and thinking skills, considered a precursor of dementia and a potential therapeutic target [4, 5]. T2DM is a risk factor for MCI [3, 6]. To date, there is no authorized diagnostic test for MCI. Aging is another factor related to cognitive impairment and aged people who are suffering from diabetes, depression, and stroke have a higher possibility of developing MCI. Previous studies uncovered that the incidence of MCI in individuals aged 70 years and older is 14–18% [7, 8]. Other studies showed that diabetic patients with MCI experiencing a greater decline in information processing speed as well as lower levels of general intelligence with loss of attention capacity than non-diabetic individuals [9]. Moreover, diabetic patients with cognitive dysfunction can further deteriorate the conditions like vision impairment, hearing loss, physical disabilities, and difficulty in performing self-care. Effective and preventive treatments are necessary at early phases of MCI with diabetics. Thus, the early detection of T2DM patients with MCI is important for patient care and treatment.

Apelin (APLN), adipocytokine, is encoded by the APLN gene, a pre-proprotein of 77 amino acids with a signal peptide in the N-terminal region. Generally, it is expressed in various organs such as the lung, kidney, liver, adipose tissue, brain, adrenal glands, heart, and human plasma [10]. APLN is expressed as an endogenous ligand for the G-protein coupled APJ receptor for some cell types (pancreatic islet cells). Besides that, it is also produced and secreted by human and mouse adipocytes [11]. In recent years significant research has been conducted to identify apelin biochemical roles and its associated signaling pathways for disease developments. A substantial number of clinical studies have been reported apelin levels in body fluids, in both healthy controls and patients with different pathologies (cardiac disease and diabetes, etc.) [12, 13]. Recent investigations point out that apelin peptide can be used as a beneficial adipokine in metabolic disorders and a promising therapeutic target for anti-obesity and anti-diabetics [14–16].

In the present study, we aimed to uncover the role of circulatory apelin in type 2 diabetes mellitus (T2DM) patients with mild cognitive impairment (MCI). We enrolled T2DM patients with mild cognitive impairments (T2DM-MCI), and T2DM patients with normal cognition (T2DM-NC), who performed the clinical assessment. We conduct a study to investigate whether serum apelin concentrations can predict MCI in type 2 diabetes in humans. The patient's demographic data and neuropsychological test results were evaluated. Blood sample analyzed for the level of apelin by enzyme-linked immunosorbent assay (ELISA). We also explored serum apelin as a biomarker and protective molecules for cognitive impairment in T2DM.

## Subjects and methods

### Study population

A total of 235 hospitalized T2DM patients were recruited from July 1, 2018 to December 31 in the Department of Endocrinology, Shanghai Pudong New Area Geriatric Hospital. The T2DM was diagnosed based on the criteria of the World Health Organization 1999 [17], and all participants had a diabetes history > 5 years. These patients were divided into two groups: T2DM patients with MCI (MCI group) and T2DM patients with normal cognition (non-MCI group). The MCI was diagnosed based on following criteria [18]: (1) a memory complaint from the patients or family members, (2) reduced memory function accessed by professional clinicians, (3) patients had normal activities of daily living and no dementia. The extent of MCI was assessed by Montreal Cognitive Assessment (MoCA) score [19]. Exclusion criteria were as follows: (1) Cognitive dysfunction due to other disorders, such as stroke, brain trauma, hypothyroidism, dementia, Parkinson’s disease, epilepsy; (2) presence of diabetic complications, such as ketoacidosis or a coma; (3) serious cerebrovascular disease history; (4) alcohol addiction, use of medication affecting circulating levels of Apelin or cognitive function within six months before enrollment; (5) acute or chronic inflammatory disease, cancer. All patients received conventional therapies, such as Acarbose.160 healthy people with matched age and gender who received physical examination in our hospital served as the control group. All subjects (patients and healthy people) provided written informed consents before sample collection. The procedure of this study was examined and approved by the Human Research Ethics Committee of Shanghai Pudong New Area Geriatric Hospital.

### Clinical data collection

The demographic data of all patients were collected, including age, sex, body mass index (BMI), education levels, height, weight, smoking and drinking. Hypertension was diagnosed based on the criteria that systolic blood pressure ≥ 140 mmHg or diastolic blood pressure ≥ 90 mmHg. Medical histories were collected, such as diabetes duration, cardiovascular disease (CVD), hyperlipidemia, retinopathy, nephropathy, and neuropathy. Peripheral blood was collected in the second morning after 12 h fasting to separate serum. The glycosylated hemoglobin (HbA1c), triglyceride (TG), total cholesterol (TC), low-density and high-density lipoprotein cholesterol (LDL-C, HDL-C), and serum creatinine were measured. The serum levels of hs-CRP, BDNF and apelin were determined by ELISA.

### Neuropsychological test

The cognitive function of all 235 T2DM patients was evaluated by MoCA. Total scores range from 0 to 30, and lower scores indicate poor cognitive function. A score of 26 or above is considered normal, and a score below 26 is defined as MCI.

### Statistical analysis

All continues variables are presented as mean ± SD, and category variables are presented as number (percentage). All statistical analysis was performed using SPSS 20.0 (SPSS Inc., Chicago, Illinois, USA). Continues variables were analyzed by T-test or Mann–Whitney U test, and categorical variables were analyzed by χ2 test. Relationships were assessed using Spearman rank correlation analysis. Multivariate regression was used to investigate the independent risk factors for MCI. *P* < 0.05 was indicated statistical significance.

## Results

### Study subject’s characteristics

The demographic and clinical parameters of the T2DM patients are shown in Table 1. There were no significant differences between MCI and non-MCI group in gender, BMI, smoking, drinking, and presence of retinopathy and neuropathy. Compared with non-MCI group, subjects in the MCI group received were older, had less education time, had longer duration of diabetes, higher incidence of CAD, hypertension, hyperlipidemia, and nephropathy. MCI patients had significantly higher serum levels of HbA1c, triglyceride, total cholesterol, LDL-C, hs-CRP, and lower serum levels of HDL-C and BDNF. The serum apelin concentration was significantly lower in T2DM patients (1.42 ± 0.35 ng/mL) compared to healthy controls 2.70 ± 0.45 ng/mL). Compared to non-MCI group (1.48 ± 0.35 ng/mL), MCI group (1.29 ± 0.31 ng/mL) had significantly lower serum levels of apelin (*P* < 0.001).

**Table 1:** Demographic and clinical characteristics of study population

Variables	Non-MCI (*n* = 162)	MCI (*n* = 73)	Z or χ2	*P* value
Age (year)	64.81 ± 4.95	66.85 ± 5.42	2.838	0.005
Male [n (%)]	89 (54.9%)	34 (46.6%)	1.411	0.235
Education (years)	11.12 ± 2.21	10.05 ± 1.81	3.903	< 0.001
BMI	25.73 ± 1.64	26.04 ± 1.45	1.399	0.163
Smoking	44 (27.2%)	26 (35.6%)	1.720	0.190
Drinking	35 (21.6%)	22 (30.1%)	1.994	0.158
Diabetes duration (years)	8.25 ± 0.97	8.83 ± 1.02	4.186	< 0.001
CVD	72 (44.4%)	47 (64.4%)	8.004	0.005
Hypertension	46 (28.4%)	32 (43.8%)	5.410	0.020
Hyperlipidemia	45 (27.8%)	31 (42.5)	4.962	0.026
Retinopathy	34 (21.0%)	20 (27.4%)	1.168	0.280
Nephropathy	39 (24.1%)	28 (38.4%)	5.036	0.025
Neuropathy	20 (12.3%)	15 (20.5%)	2.671	0.102
HbA1c (%)	7.97 ± 0.67	8.18 ± 0.65	2.233	0.027
TG (mmol/L)	2.64 ± 0.55	2.79 ± 0.43	2.094	0.037
TC (mmol/L)	6.15 ± 0.44	6.35 ± 0.54	2.909	0.004
LDL-C (mmol/L)	3.54 ± 0.39	3.70 ± 0.47	2.716	0.007
HDL-C (mmol/L)	1.80 ± 0.31	1.66 ± 0.31	3.132	0.002
Creatinine (μmol/L)	72.13 ± 8.44	75.68 ± 7.60	3.074	0.002
Hs-CRP (ng/mL)	2.12 ± 0.41	2.25 ± 0.28	2.961	0.003
BDNF (mIU/L)	9.09 ± 1.64	8.42 ± 1.19	3.556	< 0.001
MoCA	28.05 ± 1.32	23.01 ± 1.35	26.845	< 0.001
Apelin (ng/mL)	1.48 ± 0.35	1.29 ± 0.31	3.987	< 0.001

*Abbreviations*: *T2DM* Type 2 diabetes, *BMI* Body mass index, *CVD* Cardiovascular disease, *HbAlc* Glycosylated hemoglobin, *TG* Triglyceride, *TC* Total cholesterol, *LDL-C* Low-density lipoprotein cholesterol, *HDL-C* High-density lipoprotein cholesterol, *hs-CRP* high sensitivity C-reactive protein, *MoCA* Montreal Cognitive Assessment

Data are expressed as means and SD for quantitative variables and expressed as cases and percentage for category variables. Mann–Whitney U test (Z), t test or χ2 test was used to test for significant differences

### Correlation analysis

In T2DM patients, serum apelin levels were negatively correlated with diabetes duration (years) (*r* = -0.130, *P* = 0.047), TG (*r* = -0.177, *p* = 0.007), TC (*r* = -0.144, *P* = 0.027), LDL-C (*r* = -0.186, *P* = 0.004), Creatinine (*r* = -0.136, *p* < 0.001), hs-CRP (*r* = -0.159, *P* = 0.015), whereas positively correlated with HDL-C (*r* = -0.147, *P* = 0.024) and serum BDNF level (*r* = 0.190, P—0.004) (Table 2).

**Table 2:** The correlations of MoCA score with clinical indicators in T2DM patients

	Apelin	
Variables	r	*P* value
Age (year)	-0.059	0.372
Education (years)	0.056	0.393
BMI	-0.068	0.297
Diabetes duration (years)	-0.130	0.047
HbA1c (%)	-0.101	0.122
TG (mmol/L)	-0.177	0.007
TC (mmol/L)	-0.144	0.027
LDL-C (mmol/L)	-0.186	0.004
HDL-C (mmol/L)	0.147	0.024
Creatinine (μmol/L)	-0.136	0.039
Hs-CRP (ng/mL)	-0.159	0.015
BDNF (ng/mL)	0.190	0.004

*Abbreviations*: *MoCA* Montreal Cognitive Assessment, *BMI* Body mass index, *HbAlc* Glycosylated hemoglobin, *TG* Triglyceride, *TC* Total cholesterol, *LDL-C* Low-density lipoprotein cholesterol, *HDL-C* High-density lipoprotein cholesterol, *hs-CRP* high sensitivity C-reactive protein

Spearman correlation was performed

### Risk factor analysis of MCI in T2DM patients

Multivariable logistic regression analysis was performed to investigate the risk factors of MCI in T2DM patients, including education levels (years), diabetes duration (years), the presence of CVD, serum HbA1c, HDL-C, creatinine, BDNF and apelin (OR = 0.304, 95%CI = 0.104–0.886; *P* = 0.029) (Table 3).

**Table 3:** Logistic multivariate regression evaluates the risk of MCI in T2DM patients

Variables	β	SE of β	*P* value	OR	95% CI
Education (years)	-0.282	0.088	0.001	0.754	0.634–0.896
Diabetes duration (years)	0.478	0.176	0.007	1.612	1.142–2.277
CVD	0.809	0.340	0.017	2.246	1.153–4.374
HbA1c (%)	0.644	0.275	0.019	1.904	1.110–3.265
HDL-C (mmol/L)	-1.345	0.572	0.019	0.261	0.085–0.799
Creatinine (μmol/L)	0.042	0.022	0.051	1.043	1.000–1.088
BDNF (ng/mL)	-0.309	0.118	0.009	0.734	0.583–0.925
Apelin (ng/mL)	-1.190	0.546	0.029	0.304	0.104–0.886

*Abbreviations*: *β* Regression coefficient, *SE* Standard error, *OR* Odds ratio, *CI* Confidence interval for odds ratio, *MCI* Mild cognitive impairment

## Discussion

Cognition is the physiological process of acquiring and understanding information through thought, experience, and making judgments accordingly. The occurrence of cognitive impairment and changes in behavior significantly decreases the quality of life (social and professional) of diabetic patients. The association of diabetes mellitus with cognitive impairment is a serious concern to physicians worldwide. Recent evidence showed that Type 2 diabetes mellitus (T2DM) increases the risk of progression of mild cognitive impairment (MCI) to dementia and Alzheimer's disease (AD) [20]. Early diagnosis of cognitive impairments can be helpful in timely treatment. This study aimed to evaluate the diagnostic value of serum apelin as a biomarker for the early detection of MCI in T2DM patients.

Adipocytokine, Apelin is an active biomolecule that believed to play a key role in the regulation of crucial biological processes in the human body. Experimental studies established that significant alterations in adipokine secretion in disease pathophysiology. Insulin can stimulate synthesis and release of apelin from adipose tissue reported previously. An increase of plasma apelin levels and m-RNA expressions in adipocytes was found in obese subjects [21]. Other studies evidence that apelin can significantly increase glucose utilization and inhibit insulin secretion [22]. Furthermore, many studies show that apelin has an important role in DM and decreased circulating apelin levels in patients with newly diagnosed and untreated T2DM [23]. People with suffering long standing diabetes (Type 1 and Type 2) without other diabetes related complication have lower working memory [24, 25].

In the present study, there were significantly higher serum levels of HbA1c, TG, TC, LDL-C, hs-CRP in T2DM patients with the MCI group as compared to the non- MCI control group. Besides that, significantly lower serum levels of HDL-C and BDNF were also found in T2DM-MCI groups. Our results are in line with previous studies.

In our study, we found that apelin serum levels in non-diabetic subjects are significantly higher than in T2DM patients’ groups. When compared both patient groups, we found that the MCI group (1.29 ± 0.31 ng/mL) had significantly lower serum levels of apelin (*P* < 0.001) as compared to the non-MCI group (1.48 ± 0.35 ng/mL). Our results pointed that serum apelin levels were negatively correlated with T2DM with MCI. We also performed multivariable logistic regression analysis to investigate independent risk factors associated with MCI and revealed that low serum apelin level is a significant independent determinant of MCI in patients with T2DM (OR = 0.304, 95%CI: 0.104–0.886, *P* = 0.029).

Our results show that the serum apelin in diabetic patients was significantly lower than that in non-diabetic controls, which is different from the results of most other studies, which showed increased serum apelin levels in type 2 diabetes mellitus [13], and in Type 2 diabetes with diabetic peripheral neuropathy [26], proliferative diabetic retinopathy [27] and coronary artery stenosis [28]. However, decreased serum apelin levels were also observed in Type 2 diabetes, especially in newly diagnosed and untreated patients [23, 29]. This can be explained by that apelin regulates insulin sensitivity and enhances brown adipogenesis in different tissues associated with diabetes [30]. In fact, insulin stimulates apelin synthesis and release in adipocytes, and plasma apelin level markedly increases in obesity associated with insulin resistance and hyperinsulinemia [31]. A hypothesis has been proposed that high apelin levels in people with type 2 diabetes might be a compensatory mechanism for decreased insulin sensitivity [32]. Considering that most patients with type 2 diabetes are thin after disease onset, resulting in reduced size of adipose tissue, which may be associated with low serum Apelin. Our study showed slight but not significant negative correlation of serum apelin with BMI of patients, and this may provide a clue about the decreased serum apelin in T2DM patients.

We proved that serum apelin could determine MCI in patients with T2DM. However, the study is limited because all study subjects were Chinese population. Further validation of the findings in other ethnic groups is needed.

## Conclusion

In this study, it is concluded that serum apelin levels significantly reduce in T2DM-MCI patients as compared to T2DM-nonMCI patients. Apelin could be serve as independent biomarker for T2DM-associated cognitive impairment for early diagnosis. It could be considered among therapeutic agents used in the prevention of cognitive impairment and in the prevention or reduction of its critical complications. Although large scale evaluation for these candidates is required, our study provide informatics insights for T2DM-MCI detection. Serum apelin can be a powerful tool for the evaluation of cognitive impairment diseases.

## Data Availability

The datasets used and/or analyzed during the current study are available from the corresponding author on reasonable request.

## Abbreviations

APLN: Apelin
T2DM: Type 2 diabetes mellitus
IR: Insulin resistance
BMI: Body mass index
TG: Triglyceride
TC: Total cholesterol
LDL-C: Low-density lipoprotein cholesterol
HDL-C: High-density lipoprotein cholesterol
MCI: Mild cognitive impairment
AD: Alzheimer's disease
CVD: Cardiovascular disease
BDNF: Brain-derived neurotrophic factor
MoCA: Montreal Cognitive Assessment
